# Cumulative incidence and risk factors for recurrence of upper tract urothelial carcinoma in patients undergoing radical cystectomy

**DOI:** 10.1002/bco2.336

**Published:** 2024-02-29

**Authors:** Ryo Yamashita, Masafumi Nakamura, Akifumi Notsu, Koiku Asakura, Kimitsugu Usui, Yuma Sakura, Hideo Shinsaka, Masato Matsuzaki, Takashi Sugino, Ryuichi Mizuno, Masashi Niwakawa, Mototsugu Oya

**Affiliations:** ^1^ Division of Urology Shizuoka Cancer Center Shizuoka Japan; ^2^ Department of Urology Keio University Tokyo Japan; ^3^ Clinical Research Center, Shizuoka Cancer Center Shizuoka Japan; ^4^ Division of Diagnostic Radiology Shizuoka Cancer Center Shizuoka Japan; ^5^ Division of Pathology Shizuoka Cancer Center Shizuoka Japan

**Keywords:** bladder cancer, classification, radical cystectomy, recurrence, upper urinary tract

## Abstract

**Objectives:**

This study aimed to evaluate the cumulative incidence of upper tract urothelial carcinoma (UTUC) recurrence and identify its risk factors in patients who underwent radical cystectomy (RC).

**Patients and methods:**

We performed RC on 385 patients between September 2002 and February 2020. After excluding 20 patients—13 with simultaneous nephroureterectomy, 6 with distal ureteral stump positivity and 1 with urachal cancer—365 patients were included in the analysis. To predict UTUC recurrence, we examined the cancer extension pattern in cystectomy specimens and categorized them into three types: cancer located only in the bladder (bladder‐only type), cancer extending to the urethra or distal ureter (one‐extension type) and cancer extending to both the urethra and distal ureter (both‐extension type). We determined hazard ratios for UTUC recurrence for each covariate, including this cancer extension pattern.

**Results:**

Of the 365 patients, 60% had the bladder‐only type, 30% had the one‐extension type and 10% had the both‐extension type. During a median follow‐up period of 72 months for survivors, UTUC recurred in 25 of the 365 patients, with cumulative incidences of 3.7% at 5 years and 8.3% at 10 years. The median interval from cystectomy to recurrence was 65 months (interquartile range: 36–92 months). In the multivariate analysis, the extension pattern was a significant predictor of UTUC recurrence. The hazard ratios for UTUC recurrence were 3.12 (95% confidence interval [CI] = 1.15–8.43, *p* = 0.025) for the one‐extension type and 5.96 (95% CI = 1.98–17.91, *p* = 0.001) for the both‐extension type compared with the bladder‐only type.

**Conclusions:**

The cancer extension pattern in cystectomy specimens is predictive of UTUC recurrence. A more extensive cancer extension in cystectomy specimens elevates the risk of subsequent UTUC recurrence. Intensive long‐term monitoring is essential, particularly for patients with the both‐extension type.

## 1 INTRODUCTION

Radical cystectomy (RC) is the gold standard surgical procedure for muscle‐invasive, non‐metastatic bladder cancer. Multifocal metachronous tumours in the upper urinary tract can develop in patients with urothelial carcinoma (UC); therefore, urologists face a challenge in determining when to end surveillance after 5 years following RC. The incidence of upper tract urothelial carcinoma (UTUC) recurrence was reported to be only 0.8%–6.4% after RC^1^, ^2^, ^3^; however, 55%–67% of patients with UTUC recurrence died because of advanced UTUC.^3^, ^4^, ^5^, ^6^, ^7^ In fact, the upper urinary tract is one of the most common sites of late recurrence (≥3 years after RC).^1^, ^8^

Recently, Weibull analysis,^9^, ^10^ which is used to estimate the instance when a patient's risk of non‐bladder cancer death exceeds their risk of recurrence, has been used to estimate the approximate follow‐up duration in each patient. The low incidence of UTUC recurrence hinders the precise statistical analysis of the optimal surveillance duration, and the model still requires prospective evaluation. Thus, it is necessary to determine the patients who are at the highest risk for UTUC recurrence.

Diverse risk factors have been reported for UTUC recurrence, such as carcinoma in situ (CIS) in the bladder, UC of the male prostatic urethra or female urethra, history of recurrent non‐muscle‐invasive bladder cancer (NMIBC), multifocal bladder cancer, tumour involvement of the distal ureter in cystectomy specimens and RC for NMIBC.^1^, ^2^, ^3^, ^6^ Considering these risk factors, we identified that the cancer extension pattern outside the bladder, that is, in the urethra or distal ureter, is one of the key factors. We hypothesized that the risk of UTUC recurrence can be estimated by focusing on the cancer extension pattern in the urethra or distal ureter in cystectomy specimens. In the current study, we investigated the cumulative incidence of UTUC recurrence and its associated risk factors, with a focus on the cancer extension patterns in cystectomy specimens.

## 2 PATIENTS AND METHODS

### 2.1 Patient selection

Between September 2002 and February 2020, we performed open RC, bilateral pelvic lymph node dissection and urinary diversion in 385 patients with bladder cancer (cT1–cT4, N0, N1, M0 or refractory CIS). After excluding 13 patients with simultaneous nephroureterectomy (Nux), 6 patients with distal ureteral stump positivity and 1 patient with urachal cancer, 365 patients were included in this study. We usually perform a biopsy of the male prostatic urethra at the 5 and 7 o'clock positions around the verumontanum to check for CIS before RC. Urinary diversion was accomplished through either an ileal conduit or an ileal orthotopic neobladder on the basis of patient preference and cancer location. We sent a distal ureteral stump that was located 1–5 cm above the bladder for intraoperative frozen section analysis (FSA); after confirming that the ureteral stump was negative, urinary reconstructions were performed. If the first FSA was abnormal (moderate‐to‐severe dysplasia or any cancer), we sent a proximal ureter for FSA until a negative ureteral margin was confirmed. Two to three courses of neoadjuvant intravenous cisplatin‐based chemotherapy were proposed for muscle‐invasive disease. Patients were counselled on the use of adjuvant cisplatin‐based chemotherapy when they had an advanced pathological stage, such as the pT3 stage or lymph node‐positive conditions. During the study period, none of the patients received immune checkpoint inhibitors as adjuvant treatment.

**FIGURE 1:**
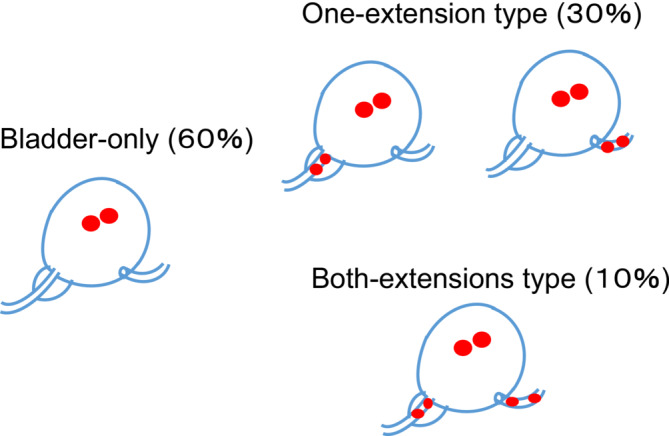
Proportions of three types among 365 cystectomy specimens based on cancer extension patterns: 217 patients (60%) had cancer located only in the bladder, 110 patients (30%) had one‐extension type (either the urethra or distal ureter) and 38 patients (10%) had both‐extension type (the urethra and distal ureter).

We evaluated the cancer location in all 365 RC specimens: cancer located only in the bladder; cancer extending to the prostatic urethra in males, to the urethra in females or to the distal ureter (one‐extension type); and cancer extending to both the urethra and distal ureter (both‐extension type) (Figure 1). We diagnosed the prostatic urethra extension in patients with cancer spreading in the prostatic urethral epithelium, but we did not include patients with bladder cancer in the bladder neck directly invading the prostatic stroma. A specimen was diagnosed as the distal ureteral‐extension type when patients had moderate‐to‐severe dysplasia or any cancer in the distal ureteral epithelium in cystectomy specimens. We did not diagnose patients as having the ureteral‐extension type when they had bladder cancer invading the outside bladder fat and directly involving the distal ureter without cancer extension on the distal ureteral epithelium.

The surveillance interval after RC was every 3–6 months for the first 2 years and then every 6 months from the third to fifth years post‐RC. Annual visits were scheduled starting 5 years after RC. Typically, we evaluate the patients using computed tomography and urinary cytology at each visit. We diagnosed a UTUC recurrence based on radiographic findings and urinary cytology.

### 2.2 Statistical analysis

We performed Fisher's exact test and the Mann–Whitney *U* test in univariate analysis. We depicted the cumulative incidence of UTUC recurrence and performed statistical analysis for comparison using Gray's test. Competing risk events were death due to any reason. The primary endpoint was to determine the cumulative incidence of UTUC and to investigate its risk factors, including cancer extension patterns in cystectomy specimens for UTUC recurrence. Multivariate analyses based on the Fine–Gray regression model were performed to determine the hazard ratio (HR) for each risk factor related to UTUC recurrence. Multivariate analysis included the reported UTUC‐related risk factors: CIS in bladder specimens, history of recurrent NMIBC, RC for NMIBC (≤pT1 or ≥pT2)^1^, ^2^, ^3^, ^6^ and our original classification system (cancer located only in the bladder, cancer extending to either the urethra or distal ureter [one‐extension type] and cancer extending to both the urethra and distal ureter [both‐extension type]). EZR software Version 1.52 (Saitama Medical Center, Jichi Medical University), which is a modified version of R Commander, was used for data analysis.^11^ *p* values < 0.05 were considered statistically significant.

## 3 RESULTS

### 3.1 Study participants

During the study period, a total of 365 RCs were analysed: 362 were diagnosed with UC, and 3 patients had pure adenocarcinoma. UC with squamous differentiation was identified in 72 patients, while glandular differentiation was observed in 33 patients. The predominant variant was the sarcomatoid variant found in 16 patients, followed by the micropapillary variant in 8 patients. The median follow‐up period for survivors was 72 months (interquartile range [IQR]: 50–113 months). Table 1 presents the patients' background data according to the cancer extension pattern. Of them, 217 (60%) exhibited cancer that was located only in the bladder, 110 (30%) displayed the one‐extension type (either to the urethra or to the distal ureter) and 38 (10%) had the both‐extension type (involving both the urethra and distal ureter), as depicted in Figure 1. Most patients were male and had undergone an ileal conduit procedure. The incidence of CIS in the resected bladder specimens varied based on the degree of cancer extension: 60% for the bladder‐only type, 77% for the one‐extension type and 92% for the both‐extension type. Similarly, the history of recurrent NMIBC varied: 12% in the bladder‐only type, 22% in the one‐extension type and 24% in the both‐extension type.

**TABLE 1:** Patient demographics and characteristics according to the cancer extension pattern (*n* = 365).

	Bladder‐only type (60%)	One‐extension type^a^ (30%)	Both‐extension type^b^ (10%)	*p* value
Number	217	110	38	
Age (years)	68	70	72	0.159
Sex, *n* (%)				<0.001
Male	174 (80%)	98 (89%)	38 (100%)	
Female	43 (20%)	12 (11%)	0 (0%)	
Body mass index, kg/m^2^	23.0	23.4	23.3	0.369
Smoking history, *n* (%)	171 (79%)	89 (81%)	34 (89%)	0.335
Diabetes mellitus, *n* (%)	42 (19%)	21 (19%)	13 (34%)	0.118
History of recurrent non‐muscle‐invasive bladder cancer	27 (12%)	24 (22%)	9 (24%)	0.041
Urinary diversion, *n* (%)				0.002
Ileal conduit	160 (74%)	93 (85%)	36 (95%)	
Neobladder	57 (26%)	17 (15%)	2 (5%)	
Perioperative chemotherapy				
Neoadjuvant	49 (23%)	21 (19%)	4 (11%)	0.223
Adjuvant	43 (20%)	20 (18%)	4 (11%)	0.429
Pathological tumour depth				0.412
≤T1	62 (29%)	39 (35%)	13 (34%)	
≥T2	155 (71%)	71 (65%)	25 (66%)	
Coexistence of carcinoma in situ, *n* (%)	130 (60%)	85 (77%)	35 (92%)	<0.001
Multifocality of bladder cancer	141 (65%)	94 (85%)	37 (97%)	<0.001
Lymph node status (positive), *n* (%)	44 (20%)	21 (19%)	7 (18%)	0.980
Cancer extension to urethra	0	47	38	
Cancer extension to distal ureter	0	63	38	
UTUC recurrence	6 (3%)	12 (11%)	7 (18%)	<0.001

*Note*: Data are median (interquartile range) unless otherwise stated.

Abbreviation: UTUC, upper tract urothelial carcinoma.

^a^ ‘One‐extension type’ refers to cancer that extends to the prostatic urethra in males, the urethra in females or the distal ureter.

^b^ ‘Both‐extension type’ refers to cancer that extends to both the prostatic urethra in males (the urethra in females) and the distal ureter.

### 3.2 Cumulative incidence and risk factors of UTUC recurrence

**FIGURE 2:**
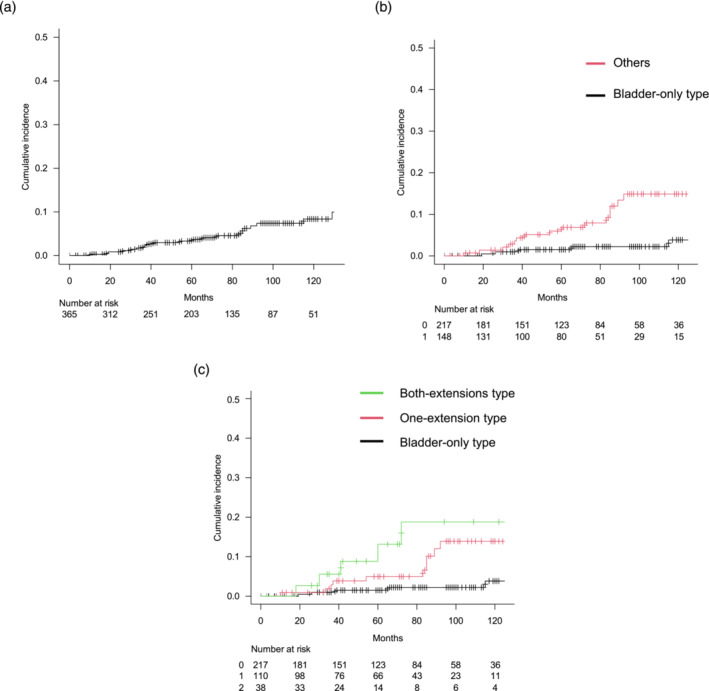
Cumulative incidence of upper tract urothelial carcinoma (UTUC) after radical cystectomy in 365 patients. (A) UTUC recurrence was observed in 25 out of 365 patients. The cumulative incidences of UTUC recurrence were 3.7% at 5 years and 8.3% at 10 years. (B) Cumulative incidences of UTUC recurrence for patients with cancer located only in the bladder (*n* = 217) were 1.5% at 5 years and 3.8% at 10 years. For patients with cancer extending to the urethra and/or distal ureter (*n* = 148), the rates were 6.8% at 5 years and 14.9% at 10 years (*p* < 0.001, Gray's test). (C) For patients with the one‐extension type (either the urethra or distal ureter, *n* = 110) and the both‐extension type (both the urethra and distal ureter, *n* = 38), the cumulative incidence rates of UTUC recurrence were 5.0% and 13.2% at 5 years and 13.9% and 18.8% at 10 years, respectively (*p* < 0.001, Gray's test).

UTUC recurrence occurred in 25 of the 365 patients. The median interval from cystectomy to recurrence was 65 months (IQR: 36–92 months). The cumulative incidence of UTUC recurrence reached 3.7% at 5 years and 8.3% at 10 years (Figure 2A). UTUC recurrence rates significantly differed between the bladder‐only type and cancer that extended to the urethra and/or distal ureter: 1.5% versus 6.8% at 5 years, respectively (Figure 2B; *p* < 0.001). Among the three classifications, patients with the both‐extension type recorded the highest cumulative incidence of UTUC recurrence at 13.2% at 5 years. Patients with the one‐extension type had an incidence of 5.0% at 5 years. Those with cancer located only in the bladder presented the lowest risk, with a UTUC recurrence of 1.5% at 5 years (Figure 2C; *p* < 0.001). In the multivariate analysis, after adjusting for the confounding factors, our classification remained significant. The HRs for UTUC recurrence were 3.12 (95% confidence interval [CI] = 1.15–8.43, *p* = 0.025) in the one‐extension type and 5.96 (95% CI = 1.98–17.91, *p* = 0.001) in the both‐extension type (Table 2).

**TABLE 2:** Univariate and multivariate analyses for recurrence of upper tract urothelial carcinoma (*n* = 365).

	Univariate	Multivariate	
	HR (95% CI)	HR (95% CI)	*p* value
History of recurrent non‐muscle‐invasive BC	3.35 (1.50–7.49)	2.40 (1.11–5.22)	0.026
Coexistence of carcinoma in situ	3.27 (0.98–10.93)	2.02 (0.55–7.30)	0.280
≥pT2 or ≤pT1 of BC (≥pT2)	0.50 (0.23–1.08)	0.78 (0.37–1.55)	0.450
Classification of radical cystectomy specimens			
Bladder‐only type	Reference	Reference	
One‐extension type^a^	4.07 (1.54–10.77)	3.12 (1.15–8.43)	0.025
Both‐extension type^b^	7.82 (2.72–22.48)	5.96 (1.98–17.91)	0.001

Abbreviations: BC, bladder cancer; CI, confidence interval; HR, hazard ratio.

^a^ ‘One‐extension type’ refers to cancer that extends to either the prostatic urethra in males (or the urethra in females) or the distal ureter.

^b^ ‘Both‐extension type’ refers to cancer that extends to both the prostatic urethra in males (the urethra in females) and the distal ureter.

### 3.3 Outcomes of patients with UTUC recurrence

Fifteen of the 25 patients underwent Nux. The pathological stages of these 15 Nux specimens were distributed as follows: ≤pT1 in seven patients, pT2 in two and pT3 in six. Nine patients (60%) exhibited concurrent CIS. Seven (46%) of the 15 patients had lymphovascular invasion. UTUC recurrences manifested in the renal pelvis for six patients, in the ureter for two and in both the ureter and renal pelvis for seven.

Ten patients did not undergo Nux due to various reasons: metastases diagnosed concurrently with UTUC (five patients), patient refusal (three patients), transitioning to another hospital (one patient) and opting for continuous surveillance (one patient). During the follow‐up period, 8 of the 25 patients succumbed to a UTUC recurrence.

## 4 DISCUSSION

The optimal duration of surveillance after RC remains controversial. In general, most bladder cancer recurrences outside the remnant urinary tract occur within 2 years after RC,^1^ owing to the intrinsic nature of rapid progression. In clinical practice, the potential for recurrence in the remnant urinary tract, specifically in the upper urinary tract and pendulous urethra, complicates urologists' decision‐making process regarding the end of surveillance. This is especially pertinent as the cumulative incidence of UTUC recurrence has been observed to increase steadily over the past 5 years.^6^ Given that recurrence can manifest in the upper urinary tract throughout a patient's lifetime, clinical guidelines recommend annual renal ultrasonography over 5 years, complemented by urinary cytology as clinically indicated.^12^ UTUC recurrences are frequently associated with a poor prognosis,^3^, ^4^, ^5^, ^6^, ^7^ emphasizing the importance of identifying patients at high risk for such recurrences.

Cancer extension to the prostatic urethra has been identified as a risk factor for UTUC recurrence.^2^, ^13^ Our study found that 23% (85/365) of patients had cancer that extended to the urethra. Ayyathurai et al. reported a similar incidence of 24% (78/320) for prostatic involvement in RC specimens.^14^ This raises questions as to why some patients had cancer that was located only in the bladder and why this extension pattern to the urethra could be a risk factor for UTUC recurrence. Li et al. reported that chromatin remodelling genes, such as *KMT2D* and *KDM6A*, were frequently mutated in the morphologically normal urothelium in patients with UC.^15^ Lawson et al. also reported these epigenetic mutations in normal urothelium, but there was extensive interindividual variation.^16^ We speculated that patients with cancer extending to the urethra or distal ureter might have broader epigenetic changes in the morphologically normal urothelium than those with the bladder‐only type. Patients with high epigenetic changes in the normal urothelium might allow a wider extension of bladder cancer and could also reflect epigenetic conditions of the remnant urothelium in the upper urinary tract, followed by a high incidence of UTUC recurrence.

The incidence of distal ureteral extension in RC specimens was high, at 101 in 365 patients (27%). The exact pathological T stage of the tumour was CIS in 58 patients, pTa in 3 patients, T1 in 3 patients, moderate–high grade dysplasia in 34 patients and T stage not stated in 3 patients. Lee et al. also reported a high incidence of intramural or juxtavesical ureteral involvement in 22 of 115 patients (19%) with RC specimens.^17^ Meanwhile, Studer et al. reported that the positive rate of FSA was very low, at 1.2%, when they cut the ureter just above the common iliac artery.^7^ In addition, only 2 (0.3%) of 805 patients with RC had recurrence at the uretero‐ileal anastomosis.^7^ Bladder cancer often extends to the intramural ureter or distal ureter; however, it rarely extends to the level of the common iliac artery. Subsequent recurrence at the uretero‐ileal anastomosis is very low when a negative distal ureteral margin is obtained. This fact confirmed that UTUC recurrence occurred de novo in the remnant urothelium in the upper urinary tract, distant from the uretero‐ileal anastomosis.

The coexistence of CIS has been identified as a potent risk factor for UTUC recurrence,^1^, ^2^, ^3^, ^6^ yet it is commonly observed in cystectomy specimens. In this study, out of 365 patients, 250 (68%) had CIS, making it an imprecise indicator of high UTUC recurrence risk (Table 2). Similarly, multifocality of bladder cancer has been cited as a prominent risk factor for UTUC recurrence.^2^, ^3^ In this study, 272 of the 365 patients (75%) demonstrated bladder cancer multifocality (Table 1), which also lacked precision in pinpointing a high risk of UTUC recurrence (Table S1). We concluded that focusing on the cancer extension patterns in cystectomy specimens can be more useful and specific when predicting UTUC recurrence after RC. Extensive extension in these specimens elevates the risk of subsequent UTUC recurrence.

UTUC recurrence is frequently associated with poor prognosis,^3^, ^4^, ^5^, ^6^, ^7^ which is partly due to challenges in early detection. Urinary cytology has low sensitivity in detecting UTUC recurrence because of degenerative changes in urine cells via the urinary‐diverted intestine^18^; therefore, invasive procedures are needed for a definitive diagnosis. However, frequent radiological examinations are not practical 5 years after RC. Fujii et al. demonstrated the high diagnostic value of sequencing urinary sediment‐derived DNA for the non‐invasive detection of UTUC.^19^ They categorized UTUC into five molecular subtypes: hypermutated, *TP53/MDMD2*, *RAS*, *FGFR3* and triple negative. This sequencing method for urine sediment‐derived DNA achieved 82.2% sensitivity and 100% specificity, outperforming urinary cytology, which exhibited only 32.9% sensitivity and 88.9% specificity. As sequencing costs continue to decrease, analysing urinary sediment samples may provide an alternative to conventional invasive diagnostic procedures for UTUC.

The limitations of this study include the single‐centre nature, limited patient population and challenges in pathologically staging UTUC recurrence. We performed only 15 Nux out of 25 cases, thus not evaluating the detailed pathological tumour stage for the remaining 10 UTUC recurrences. Furthermore, we submitted the first distal ureteral stump 1–5 cm from the bladder. The distance from the bladder to the ureteral stump varied slightly, depending on the surgeon. We did not measure the exact length of the ureter from the bladder. Therefore, the cancer incidence of the distal ureter might differ if the ureteral length were measured more precisely.

## 5 CONCLUSIONS

In summary, patients with both‐extension type should be closely monitored over 5 years after RC. This extension pattern in cystectomy specimens should always be considered when predicting UTUC recurrence.

## AUTHOR CONTRIBUTIONS

All named authors contributed to the creation of this manuscript.

## CONFLICT OF INTEREST STATEMENT

The authors have no conflicts of interest to declare.

## Supporting information


**Table S1.** Univariate and mutivariate analyses including the factor of multifocality for recurrence of upper tract urothelial carcinoma (n = 365).
