# Supplementary material for: Cumulative incidence and risk factors for recurrence of upper tract urothelial carcinoma in patients undergoing radical cystectomy
Source: BJUI Compass. 2024 Feb 29;5(5):483–9. doi: 10.1002/bco2.336 (PMC11090764; doi:10.1002/bco2.336)
Supplement: Supplementary file 1 — Table S1. Univariate and mutivariate analyses including the factor of multifocality for recurrence of upper tract urothelial carcinoma (n = 365). [file BCO2-5-483-s001.docx]

**Table S1.** Univariate and mutivariate analyses including the factor of multifocality for recurrence of upper tract urothelial carcinoma (n = 365).

|  | Univariate | Multivariate | |
| --- | --- | --- | --- |
|  | HR (95% CI) | HR (95% CI) | p value |
| History of recurrent non-muscle invasive BC | 3.35 (1.50–7.49) | 2.12 (0.96–4.69) | 0.061 |
| Multifocality of BC | 4.43 (1.01–19.55) | 2.16 (0.44–10.40) | 0.340 |
| ≥pT2 or ≤pT1 of BC (≥pT2) | 0.50 (0.23–1.08) | 0.72 (0.35–1.48) | 0.380 |
| Classification of radical cystectomy specimens:  - Bladder-only type | reference | reference |  |
| - One-extension type | 4.07 (1.54–10.77) | 2.96 (1.11–7.89) | 0.030 |
| - Both-extensions type | 7.82 (2.72–22.48) | 5.53 (1.92–15.95) | 0.001 |

|  | Univariate | |
| --- | --- | --- |
|  | HR (95% CI) | p value |
| History of recurrent non-muscle-invasive BC | 3.71 (1.19–11.57) | 0.024 |
| Coexistence of carcinoma in situ | 3.52 (0.45–27.22) | 0.230 |
| ≥pT2 or ≤pT1 of BC (≥pT2) | 0.58 (0.20–1.70) | 0.330 |
| Classification of radical cystectomy specimens:  - Bladder-only type | reference |  |
| - One-extension type | 6.03 (1.52–23.85) | 0.010 |
| - Both-extensions type | 8.17 (1.77–37.61) | 0.007 |

|  | Univariate | Multivariate | |
| --- | --- | --- | --- |
|  | HR (95% CI) | HR (95% CI) | p value |
| History of BCG treatment | 3.22 (1.42–7.27) | 2.56 (1.13–5.79) | 0.023 |
| Coexistence of carcinoma in situ | 3.27 (0.98–10.93) | 2.07 (0.57–7.43) | 0.260 |
| ≥pT2 or ≤pT1 in bladder cancer (≥pT2) | 0.50 (0.23–1.08) | 0.66 (0.31–1.40) | 0.290 |
| Classification of radical cystectomy specimens:  - Bladder-only type | reference | reference |  |
| - One-extension type | 4.07 (1.54–10.77) | 3.34 (1.25–8.95) | 0.016 |
| - Both-extensions type | 7.82 (2.72–22.48) | 5.68 (1.79–18.01) | 0.003 |
